# The Use of Immersive Virtual Reality in Sensory Sessions on an Older Peoples Mental Health Ward: Service Evaluation of Feasibility and Acceptability

**DOI:** 10.1192/bjo.2025.10471

**Published:** 2025-06-20

**Authors:** Ciju Benjamin, Felix Clay

**Affiliations:** 1 Cambridgeshire and Peterborough NHS Foundation Trust, Cambridge, United Kingdom; 2 Norfolk and Suffolk NHS Foundation Trust, Norwich, United Kingdom

## Abstract

**Aims:** Immersive virtual reality has the potential to give people admitted on inpatient ward settings a break from these limited environments. This service evaluation reviewed the use of immersive virtual reality relaxation activities as a part of routine occupational therapy sensory sessions in an older people’s inpatient mental health ward for dementia and functional conditions. We assessed acceptability and feasibility by reviewing user experience and therapeutic engagement in terms of relaxation, engagement and interaction.

**Methods:** This evaluation was approved by Cambridge and Peterborough Foundation Trust Quality Improvement panel and assessed routinely collected data from 32 users (9 from dementia unit, 23 from functional unit) across a total of 158 sessions visiting nature scenes on a Pico 4 headset across an 11 month period in 2023. Demographic information included age, gender, mental health and other diagnoses, reason for admission, regular medication and legal status. Occupational therapy notes were assessed for subjective experience, positive and negative effects, interaction, therapy engagement, preferred scene, duration and repeat use.

**Results:** Average user age was higher on the dementia unit vs functional unit (77.5 vs. 74.5 years). Primary mental health diagnosis was a dementia subtype for most service users on the dementia unit (6/9) compared with a wider variety of diagnoses on the functional unit (depression or bipolar disorder 7/23 each; schizophrenia, alcohol related or delusional disorder 2/23 each; obsessive compulsive disorder, dementia, or personality disorder 1/23 each). Most service users on the dementia unit and functional unit (96% vs. 97%) reported a positive experience and therapists reported relaxation in most users (88% vs. 83%). Duration of use was shorter on the dementia unit compared with the functional unit (mean 5 minutes 36 seconds vs. 7 minutes 42 seconds) and repeat use was also lower (2.7 sessions vs. 5.4 sessions). No serious adverse effects were noted and <3% sessions resulted in any side effects.

**Conclusion:** This service evaluation demonstrates feasibility and acceptability of immersive virtual reality relaxation activities as part of routine occupational therapy sensory sessions on an older people’s mental health ward supporting services users with a wide variety of mental health diagnoses. Relaxation and calming were reported by therapists with no serious adverse effects. Many patients chose to return to the headset on multiple occasions especially on the functional unit where they completed longer sessions compared with the dementia unit. Research is planned into potential benefits for anxiety, stress reduction, sleep and medication use.

